# Oral PSORI-CM01, a Chinese herbal formula, plus topical sequential therapy for moderate-to-severe psoriasis vulgaris: pilot study for a double-blind, randomized, placebo-controlled trial

**DOI:** 10.1186/s13063-016-1272-x

**Published:** 2016-03-16

**Authors:** Dan-Ni Yao, Chuan-Jian Lu, Ze-Huai Wen, Yu-Hong Yan, Mei-Ling Xuan, Xiao-Yan Li, Geng Li, Ze-Hui He, Xiu-Li Xie, Jing-Wen Deng, Xin-Feng Guo, Ai-Hua Ou

**Affiliations:** Department of Dermatology, Guangdong Provincial Hospital of Chinese Medicine, No. 111 Dade Road, Guangzhou, 510120 China; Guangdong Provincial Academy of Chinese Medical Sciences, Guangzhou, 510120 China; Guangdong Provincial Key Laboratory of Clinical Research on Traditional Chinese Medicine Syndrome, Guangzhou, 510120 China; Key Unit of Methodology in Clinical Research, Guangdong Provincial Hospital of Chinese Medicine, Guangzhou, 510120 China; National Centre for Design Measurement and Evaluation of Clinical Research, Guangzhou University of Chinese Medicine, Guangzhou, 510405 China; Team of standardization of Chinese Medicine, Guangdong Provincial Hospital of Chinese Medicine, Guangzhou, 510120 China

**Keywords:** Psoriasis vulgaris, pilot randomized controlled trial, PSORI-CM01, Chinese herbal medicine, topical sequential treatment

## Abstract

**Background:**

To provide evidence that the Chinese herbal medicine (CHM) PSORI-CM01 combined with Western medicine reduces the relapse rate of psoriasis vulgaris (PV), we plan to conduct a large-scale randomized control trial (RCT). In order to improve and perfect the RCT, this pilot study was designed to determine the feasibility and the potential of a modified protocol for the full-scale RCT.

**Methods:**

Eligible patients with psoriasis vulgaris (PV) were enrolled into a randomized comparison in which all subjects received topical sequential therapy and PSORI-CM01 or placebo for 12 weeks. The primary outcome measure was the relapse rate. Treatment response was computed from Psoriasis Area and Severity Index (PASI), body surface area (BSA), and Dermatology Life Quality Index (DLQI). The secondary outcome measures included time to relapse, time to onset, rebound rate, PASI score, pruritus scores on the Visual Analog Scale (VAS), BSA, DLQI and SF-36 (short form health survey), and incidence of serious adverse events (SAEs).

**Results:**

Six of 7 (86 %) subjects reached the PASI-50 in the CHM group compared with nine of 10 (90 %) in the placebo group during the treatment period. Among the subjects who reached PASI-50, one out of six subjects (17 %) relapsed in the CHM group during the treatment period compared with six out of nine patients in the placebo group (67 %). No subjects met the rebound criteria. Changes to baseline in the PASI scores were not significantly different between the two groups (*t* = 1.764, *P* = 0.098).

**Conclusion:**

Oral PSORI-CM01 combined with topical sequential treatment showed a smaller recurrence rate (*P = 0.118*) than placebo combined with the same topical therapy for moderate-to-severe PV in this pilot study.

**Trial registration:**

Chinese Clinical Trial Registry (http://www.chictr.org.cn/searchproj.aspx) ChiCTR-TRC-13003233; date of registration: 15 April 2013.

**Electronic supplementary material:**

The online version of this article (doi:10.1186/s13063-016-1272-x) contains supplementary material, which is available to authorized users.

## Background

Psoriasis is a chronic skin disease characterized by disfiguring scaling and erythematous plaques that may be painful or often severely pruritic and may cause many quality of life (QOL) issues [1]. Psoriasis vulgaris (PV) is the most common form and is categorized as “mild, moderate-to-severe” according to the rules of ten [2]. A quarter of those affected have moderate-to-severe disease for which treatment options include topical treatment, systemic treatment, or phototherapy. Currently, no curative treatment is available. Due to its chronic nature, frequent relapses, and high impact on QOL, patients often require long-term treatment. However, most of the therapies are associated with side effects and not appropriate for long-term use. Therefore, the goal of treating psoriasis is to maintain the success of the treatment as far as possible with fewer side effects.

Chinese herbal medicine (CHM) has a history of successful use in treating PV in China. Meta-analyses of clinical trials suggest clinical benefit for at least some CHM treatments [3–5]. Evidence showed that a combination of CHM and Western medicine relieved symptoms and reduced PASI scores with fewer side effects and toxicity [6, 7]. However, no evidence of CHM for reducing the relapse of PV is available.

PSORI-CM01, a Chinese herbal formula, has been used for more than 20 years in Guangdong Provincial Hospital of Chinese Medicine (GPHCM) in China to reduce the recurrence rate of PV. We determined the feasibility of conducting a multicenter, randomized controlled trial (RCT) to evaluate the effectiveness and safety of PSORI-CM01 combined with topical therapy compared with placebo for PV, focusing on the relapse rate and reduction of PASI. To this end, we conducted a feasibility pilot study, which was registered on 21 August 2012 (ChiCTR-TRC-12002447), comparing PSORI-CM01 plus calcipotriol betamethasone ointment therapy with placebo plus the topical therapy. The results of the CHM group showed 50 % improvement in PASI earlier than the placebo group, with a longer maintenance efficacy. However, the study had limitations associated with the interventions and study period. The trial did not register good compliance from 6 weeks to the end mainly due to the rebound effect after 4 weeks of the calcipotriol betamethasone ointment therapy. One patient was reported to manifest pustular psoriasis after 4 weeks of calcipotriol betamethasone topical treatment as an adverse drug reaction. Therefore, topical sequential treatment was needed to reduce the hazards associated with the rebound and adverse effect of topical corticosteroids.

Topical sequential therapy with a combination of calcipotriol and calcipotriol betamethasone was significantly superior to the use of each therapy alone from a pharmacoeconomic standpoint according to the Guidelines for the Treatment of PV [8]. To minimize the adverse effects and maximize compliance, we modified the protocol by adding 8 weeks of maintenance therapy with calcipotriol after 4 weeks of calcipotriol betamethasone ointment, based on Guidelines and evidences for moderate to severe psoriasis with BSA less than 30 % [8, 9]. We designed a run-in period before enrollment and used urea ointment and cetirizine hydrochloride as rescue medicine when skin itchiness affected the patients’ QOL during the whole study. The modified protocol was registered in April 2013 (ChiCTR-TRC-13003233) and published in July 2014 [10]^.^

The objective of this pilot study was to determine the feasibility of the modified protocol and assess the potential for a full RCT. The primary outcome measure was the incidence of relapse during the study.

## Methods

### Design and eligibility

This pilot study was designed as a randomized, double-blind, placebo-controlled trial to evaluate the efficacy of oral PSORI-CM01 plus topical sequential treatment compared with placebo plus topical sequential treatment for moderate-to-severe PV. A randomized number was generated by computer with a randomized block method in which the block size was four, in a ratio of 1:1, created by SAS 9.2 software (SAS Institute Inc., Cary, USA) and performed by the Key Unit of Methodology in Clinical Research (KUMCR) of GPHCM. The result of the randomized allocation was concealed in an opaque envelope and was also performed by the KUMCR of GPHCM. The participants, paramedics, investigators, outcome assessors, statistician and other staff who help dispense medications were all blinded to the allocation. The study protocol was approved by the Institutional Ethics Committee of GPHCM (Ethics Statement NO: B2012-53-03, approved on 16 August 2013), and all patients provided written informed consent prior to initiation of study.

### Setting

**Fig. 1:**
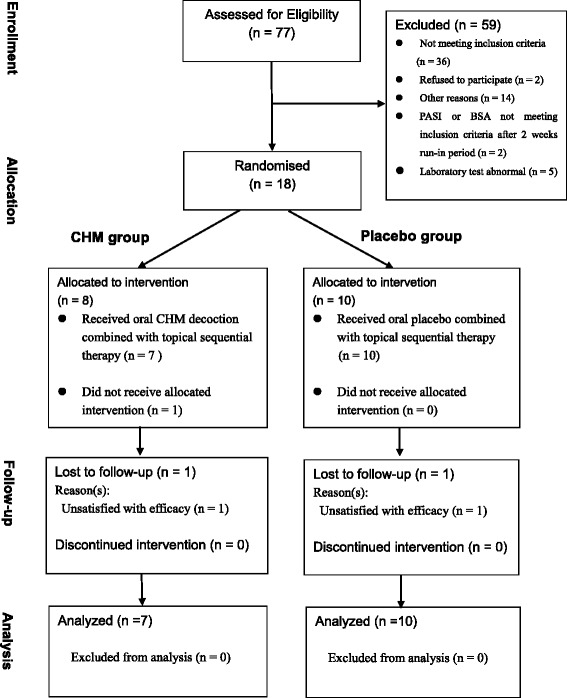
CONSORT diagram of the pilot study. CHM, Chinese herbal medicine; PASI, Psoriasis Area Severity Index; BSA, body surface area

Prior to randomization, all patients had a run-in period of 2 weeks before receiving treatment. Before the run-in period, all patients agreed to cease topical corticosteroid therapy, vitamin D analogs, keratolytics, and coal tar or CHM for 2 weeks, and phototherapy and systemic therapies for 4 weeks to meet the criterion. Only urea cream was permitted during the run-in period. After this period, subjects were screened again according to inclusion and exclusion criteria. Eligible subjects were assigned according to their sequential numbers, which corresponded to a random number and then were randomized to one of the two groups (see Fig. 1 for the CONSORT flowchart).

### Patients

Patients were recruited by Dermatologist or investigators mostly from dermatology outpatients in the GPHCM or through local newspapers or posters. A few patients spontaneously contacted the trial center. Participants were diagnosed with PV by dermatologists. The inclusion criteria were as follows: 1) moderate to severe psoriasis (PASI > 10 or BSA > 10 %, PASI < 30 and BSA < 30 %); 2) written/signed informed consent; 3) age: 18 to 65 years.

The exclusion criteria were as follows: 1) guttate psoriasis, inverse psoriasis, or psoriasis exclusively associated with the face; 2) current (or within 1 year) pregnancy or lactation; 3) current clinically important anxiety or depression with the Self-rating Anxiety Scale (SAS) > 50 or the Self-rating Depression Scale (SDS) > 53, or with other psychiatric disorders; 4) history of uncontrolled cardiovascular, respiratory, digestive, urinary, or hematologic disease; 5) history of known cancer, infection, electrolyte imbalance, acid-base disturbance, and calcium metabolic disorder; 6) abnormal serum calcium level (Ca2+ > 2.9 mmol/L or < 2 mmol/L); 7) allergy to any medicine or ingredients used in this study; 8) current registration in other clinical trials or participation within a month; 9) topical treatments (that is, corticosteroids or retinoic acid) within 2 weeks; 10) systemic therapy or phototherapy (ultraviolet radiation B, UVB) and psoralen combined with ultraviolet A (PUVA) within 4 weeks or biological therapy within 12 weeks; 11) acute progression of psoriasis, and erythroderma tendency; or 12) systemic treatment prescribed by doctors.

### Intervention

The topical sequential treatment in the study was calcipotriol betamethasone ointment (DaivobetR Ointment, containing calcipotriene 50 μg/g and betamethasone 0.5 mg/g as dipropionate; LEO Laboratories Ltd, Dublin, Ireland) once daily for 4 weeks, followed by calcipotriol ointment (DovonexR cream; LEO Laboratories Ltd, Dublin, Ireland) once daily for 8 weeks. Patients were randomized (1:1) to receive either a PSORI-CM01 decoction or placebo orally. The PSORI-CM01 decoction, composed of Radix Paeoniae Rubra, Rhizoma Curcumae, Sarcandra, Radix glycytthizae, Fructus Mume, Radix Arnebiae, and Rhizoma Smilacis Glabrae, was decocted and packaged into vacuum packs by the pharmaceutical department of Chinese herbal medicine of GPHCM (100 mL/pack). The matching placebo, the main ingredients, of which were maltodextrin, lactose, and natural edible pigment, was similar to the PSORI-CM01 decoction in appearance, weight, and taste, and was also manufactured by the pharmaceutical department of Chinese herbal medicine of GPHCM. The quality control of the decoction had been accredited by the Guangdong Provincial Laboratory using liquid chromatography coupled with an LTQ Orbitrap mass spectrometer [11]. Patients were administered the PSORI-CM01 decoction or placebo single pack twice a day after meals.

The treatment duration was 12 weeks. Urea ointment was used as the basic concomitant treatment in the run-in period, according to doctors’ choice. In case of a serious itch, cetirizine hydrochloride (10 mg/day) was the rescue drug, based on doctors’ advice. The study flowchart is illustrated in Fig. 1.

### Outcome measures

The primary outcome measure in this pilot study was to evaluate the efficacy of a combination of PSORI-CM01 and topical sequential treatment to reduce the relapse rate of patients during the treatment period. Relapse was defined as the loss of 50 % of PASI improvement from baseline in patients who achieved treatment success (at least 50 % improvement in PASI score from baseline) [12, 13]. Relapse was determined by evaluating the proportion of subjects who lost PASI-50 response attained during the treatment period.

Secondary outcome measures in the study were as follows: (i) the relapse time interval, (ii) time to onset, (iii) rebound rate, (iv) improvement in PASI, (v) pruritus scores on the Visual Analog Scale (VAS), (vi) improvement in BSA for PV, (vii) improvement in DLQI and SF-36 (the MOS item short form health survey), and (viii) incidence of serious adverse events (SAEs).

The PASI and BSA were assessed by investigators every week during the first 4 weeks and every 2 weeks during the rest of the treatment period. Meanwhile, patients were required to report VAS scores and the emergence of altered condition at any time during study period.

DLQI and SF-36 were calculated every 4 weeks. Other outcome measures were calculated during patient visits.

Laboratory tests including complete blood count, urinalysis, hepatic and renal functions, and electrocardiogram were monitored and recorded at baseline and 12 weeks after treatment.

### Statistical analysis

Data were processed with PASW Statistics 18.0 (IBM SPSS Inc, Armonk, New York, USA). Descriptive analysis was used to characterize patients’ clinical demographics. The primary outcome was compared 12 weeks after treatment between both groups using the Chi-square test (or Fisher’s exact test). Secondary outcomes were compared between both groups by the end of treatment using *t*-tests, the Mann-Whitney test, and Fisher’s exact test. Two-tailed *P* values < 0.05 were considered statistically significant. The last observation carried forward was used to evaluate data with an intention-to-treat analysis. Data were presented as mean and standard deviation.

Since it was a pilot study instead of a hypothesis test, it is inappropriate to make any inferences or state findings of efficacy [14]. The aim of this pilot trial was to test the practicality of the trial design, and data were only analyzed to alert the investigators to issues for testing in the planned larger-scale trial.

### Sample size

For the large-scale study, we calculated sample size based on White’s study [9] in the protocol [10]. Sample size calculations are not a prerequisite for pilot studies [15]. Following recommendations for pilot trials, 18 patients were recruited at baseline to test the practicality of the trial design.

## Results

### Patients

In this pilot study, 77 patients were recruited for 2 months, and 18 (8 in CHM group vs 10 in placebo group) of them were enrolled from the outpatient department at GPHCM. However, one of the randomized patients in the CHM group did not receive the allocated intervention and had no data from baseline; therefore 17 patients were analyzed. Of these, 15 (88 %) completed the study at week 12. Two patients who discontinued did not show satisfactory efficacy. The majority of subjects were male (89 %), with a mean age of 43 years (minimum to maximum: 24 to 62 years). A summary of baseline demographic information is provided in Table 1. The mean PASI score was 13.19 in the CHM group compared with 11.67 in the placebo group at baseline. The groups were similar in size, sex, age, and mean PASI at baseline (Table 1).

**Table 1:** Patients’ baseline characteristics

	CHM group	placebo group
Number of subjects	7	10
Sex		
Male	7	8
Female	0	2
Age, years (Mean, SD)	45.43, 11.84	41.60, 13.24
Min-Max	29 to 59	24 to 62
PASI, (Mean, SD)	13.19, 3.66	11.67, 4.17
(min-max)	(10.1 to 20.4)	(6.5 to 17.6)
Disease duration, months (minimum to maximum)	252 to 601	21 to 192
(Mean, SD)	144.57, 73.77	86.20, 54.02
Job, n (%)		
Worker	2 (29)	1 (10)
Farmer	2 (29)	1 (10)
Cadres	0 (0)	1 (10)
Clerk	1 (14)	5 (50)
Retired or unemployed	0	0
Freelance work	1 (14)	2 (20)
Others	1 (14)	0
Marital status, n(%)		
Married	5 (63)	6 (60)
Unmarried	3 (38)	4 (40)
Education, n(%)		
Illiterate	1 (14)	1(10)
Middle school	2 (29)	0
High school	3 (43)	2 (20)
College graduate	1 (14)	7 (70)
Family history, n(%)	0	
With	0	5 (50)
Without	7 (100)	5 (50)
BMI, (Mean, SD)	22.76, 3.70	24.16, 2.67
Stage, n(%)		
Active	0	0
Stable	7 (100)	10 (100)
Regressive	0	0

*CHM* Chinese herbal medicine, *BMI* body mass index, *PASI* Psoriasis Area Severity Index, *SD* standard deviation

### Efficacy

#### Primary outcome measure

Recurrence was the primary outcome. During the treatment period, six of the seven (86 %) subjects in the CHM group reached PASI-50 and one of them (17 %) relapsed during the treatment period after attaining PASI-50, whereas five maintained PASI-50 at the end of 12 weeks. However, nine of 10 (90 %) subjects in the placebo group reached PASI-50, and six of them relapsed (67 %), whereas three attained treatment success (maintaining PASI-50). Relapse was 17 % in the CHM group versus 67 % in the placebo group, with no statistically significant difference in the incidence of relapse between the two groups (odds ratio: 0.10; 95 % CI: 0.008, 1.288; *P* = 0.119) (Table 2).

**Table 2:** Relapse rate and rebound rate

Group		Treatment success		relapse		rebound	
	*n*	PASI-50	Never PASI-50	relapse	No relapse	rebound	No rebound
CHM	7	6 (86 %)	1 (14 %)	1 (17 %)	5 (83 %)	0 (0 %)	6 (100 %)
Placebo	10	9 (90 %)	1 (10 %)	6 (67 %)	3 (33 %)	0 (0 %)	9 (100 %)
Total	17	15	2	7	8	0	15

*CHM* Chinese herbal medicine

PASI-50, PASI score decreases more than 50 % from baseline

Relapse, loss of 50 % of PASI improvement from baseline in patients who have achieved treatment success (at least 50 % improvement in PASI score from baseline)

Rebound, defined only for patients who achieved PASI-50, occurred when the improvement in the PASI score increased up to 25 % from the baseline PASI score

There was no difference in the relapse rate between the two groups by Chi-square (odds ratio: 0.10; 95 % CI: 0.008, 1.288; *P* = 0.119)

#### Secondary outcome measure

The relapse time interval was calculated from the time patients achieved PASI-50 until its initial loss. In this pilot study, the mean relapse time interval in the CHM group was 6.00 weeks (only one patient relapsed) compared with 4.42, 2.44 (mean, SD) weeks in the placebo group (see Table 3).

**Table 3:** Relapse time interval, Time to onset

Assessment	Group	*n*	Min	Max	$\overline{x}\pm s$	*t*	*P*
Relapse time interval	CHM	1	6 weeks	6 weeks	6.00, 0.00	NA	NA
	Placebo	6	1 weeks	8 weeks	4.42, 2.44		
Time to onset	CHM	6	1 weeks	7 weeks	3.33, 2.25	1.802	0.095
	Placebo	9	1 weeks	4 weeks	1.78, 1.09		

*CHM* Chinese Herbal Medicine

Relapse time interval was determined from the time patients achieved PASI-50 until its initial loss

Time to onset was determined by the time when the PASI score decreased more than 50 % for the first time from baseline

*NA* not applicable

Time to onset was determined as the time when the PASI score decreased more than 50 % for the first time from baseline. The mean onset time of this pilot study was 3.33, 2.25 weeks (minimum to maximum: 1 to 7 weeks) in the CHM group versus 1.78, 1.09 weeks (minimum to maximum: 1 to 4 weeks) in the placebo group (Table 3).

Rebound rate, that is, the proportion of patients with PASI scores rebounding, was defined only for patients who achieved PASI-50 and occurred when the improvement in the PASI score increased up to 25 % from the baseline PASI score. No patients reached rebound in the study, and therefore, the rebound rate was 0 % in both groups (see Table 2). Group comparisons of outcomes above were not analyzed due to an insufficient number of participants in this trial.

**Fig. 2:**
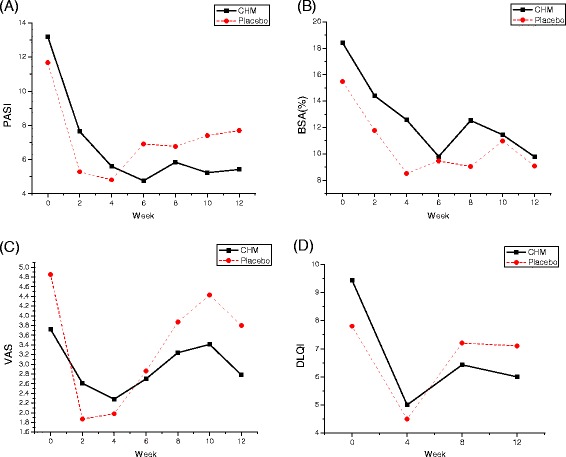
Psoriasis Areas Severity Index (PASI) score, body surface area for psoriasis (BSA) score, Visual Analog Score (VAS), and Dermatology Life Quality Index (DLQI) score in the Chinese herbal medicine (CHM) and placebo groups. (**a**) PASI scores; (**b**) BSA scores; (**c**) VAS scores; and (**d**) DLQI scores. Comparison of changes of PASI (*t* = 1.764, *P* = 0.098), BSA (*t* = 0.523, *P* = 0.610), VAS (*t* = -0.079, *P* = 0.938), and DLQI (*t* = 0.845, *P* = 0.411) after treatment between the two groups. *P* < 0.05 indicated a significant difference between groups

**Fig. 3:**
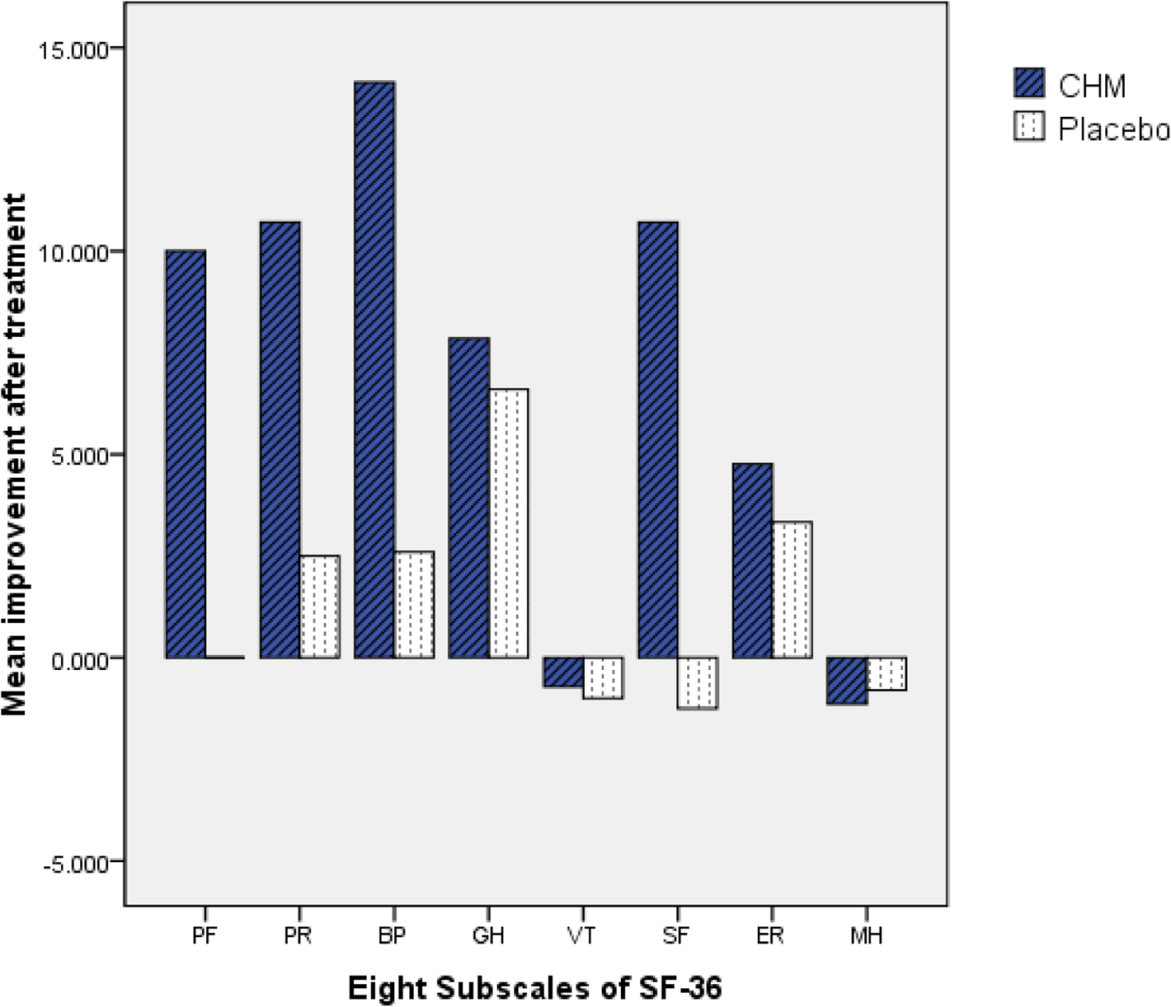
Mean improvement in Short Form-36 Health Survey (SF-36) subscale scores at week 12 in both groups. CHM, Chinese herbal medicine; PF, physical function (limitations in physical activities because of health problems); PR, physical role (limitations in usual role activities because of physical health problems); BP, bodily pain; GH, general health perceptions; VT, vitality (energy and fatigue); SF, social function (limitations in social activities because of physical or emotional problems); ER, emotional role (limitations in usual role activities because of emotional health problems); MH, mental health. No significant differences were observed between the groups in any subscale scores. A comparison of changes of subscale scores after treatment between two groups: *P* < 0.05 indicated significant difference between groups. The statistical results of the subscale scores are as follows: PF (*Z* = -1.742, *P* = 0.081), PR (*Z* = -0.875, *P* = 0.391), BP (*Z* = -1.913, *P* = 0.056), GH (*t* = 0.171, *P* = 0.868), VT (*Z* = -0.298, *P* = 0.766), SF (*Z* = -1.086, *P* = 0.278), ER (*Z* = 0.00, *P* = 1.00), MH (*Z* = -0.451, *P* = 0.652)

The changes of PASI, VAS, BSA, and DLQI scores in the CHM group after treatments seemed much more than the placebo group from week 4 to week 12 (Fig. 2). At the end of 4 weeks, the PASI, BSA, VAS, and DLQI curves of both groups showed similar tendency to decline. From 6 to 12 week, PASI, and VAS scores were lower in the CHM group than in the placebo group (Fig. 2). It was interesting that the PSORI-CM01 decoction not only improved lesions, but also improved health-related quality of life (HRQOL) in psoriasis patients, as demonstrated by improvements of the DLQI scores (see Fig. 2) and SF-36 (see Fig. 3).

Improvements of the DLQI score in the study were 3.43, 7.76 in the CHM group compared with 0.70, 6.00 in the placebo group (*t* = 0.845, *P* = 0.411) (Fig. 2). The following eight subscales were assessed with 36 items of the SF-36: PF, physical function (limitations in physical activities because of health problems); PR, physical role (limitations in usual role activities because of physical health problems); BP, bodily pain; GH, general health perceptions; VT, vitality (energy and fatigue); SF, social function (limitations in social activities because of physical or emotional problems); ER, emotional role (limitations in usual role activities because of emotional health problems); and MH, mental health. The CHM group tended to have a higher mean of altered scores in the subscales, which suggested the QOL improved more than in the placebo group except for MH (mental health) (see Fig. 3).

As indicated in the tables, differences in the PASI, BSA, VAS, and DLQI scores were not significant between the CHM group and the placebo group after treatment.

#### Safety

No serious adverse events (SAEs) or abnormal laboratory test results were observed during the entire study period. Four patients (57 %, 4/7) exhibited adverse events in the CHM group, and three (30 %, 3/10) in the placebo group, which was not significantly different between the two groups. Three patients reported lower limb erythema and burning heat sensation, and one also experienced edema while using the calcipotriol ointment. One of them was in the CHM group, whereas two were in the placebo group. The remaining side effects such as contact dermatitis, hypertension, dizziness, and common cold were limited to a single case in both groups.

## Discussion

This pilot trial was successful in patient recruitment, randomization, and retention and assessment. The trial design was feasible and acceptable to both patients and investigators. Seventy-seven patients were recruited from the outpatient department within 3 months. Of the 25 screened participants, 18 were enrolled. One of the 18 randomized patients did not receive the allocated intervention. Therefore, only 17 patients were included the study. Two participants (11 %) withdrew from the trial. Treatment adherence was better than that in the first pilot study (registered on 21 August 2012 (ChiCTR-TRC-12002447), possibly because this pilot study was modified by adding a run-in period and sequential topical therapy. Fifteen patients who completed the trial returned with good therapeutic adherence. All of the outcome measures were assessed at every time point.

As far as we know, the pilot study is the first to explore PSORI-CM01, a Chinese herbal formula, combined with topical sequential treatment to reduce the recurrence rate of PV. Psoriasis is often characterized by acute episodes and remission phases, requiring chronic therapy. The results from our study suggested that the addition of PSORI-CM01 to topical sequential treatment was an effective treatment for moderate to severe psoriasis. It reduced the rate of relapse. Maintaining the treatment success and reducing the relapse are the main goals of disease management. The results of this pilot study showed that PSORI-CM01 was a useful addition to conventional therapy.

The addition of PSORI-CM01 to topical sequential treatment not only improved lesion conditions but also helped QOL. Outcome measures such as PASI, BSA, and VAS scores showed a declining pattern in both groups after treatment. The PASI and VAS scores in the CHM group seemed to improve more than in the placebo group, which demonstrated long-term treatment success. The PSORI-CM01 decoction relieved itch in rat models [16]. Data also suggested that the CHM group had lower mean VAS scores than the placebo group at week 12. Outcome measures such as DLQI and SF-36 also suggested that PSORI-CM01 helped to improve the QOL more than placebo and maintained treatment success similar to PASI scores. The mean improvements in the DLQI scores and SF-36 were greater in the CHM group than in the placebo group. Therefore, the addition of PSORI-CM01 reduced relapse, probably by clearing lesions, relieving the itch, and improving the QOL.

CHM has been used for a long time in China to control psoriasis clinically. However, the limitation of its broader use might be related to its slower action. Topical ointment cleared lesions rapidly but without lasting effects. Therefore, a combination of CHM and topical sequential therapy to treat psoriasis is a new option to clear lesions faster and maintain success longer than individual topical therapies [17].

A systematic review previously evaluated the effects of oral Chinese herbal medicine combined with pharmacotherapy for PV compared with pharmacotherapy [4]. The results showed the benefit of the combination. The most frequently used herbs were *Rehmannia glutinosa* root (Sheng di), *Angelica sinensis* root (Dang Gui), *Smilax glabra* root (Tu Fu Ling), *Paeonia veitchii* root (Chi Shao), *Salvia miltiorrhiza* root (Dan Shen), and *Lithospermum erythrorhizon* root (Zi Cao). Most of them were compositions of the prescription in PSORI-CM01 formula.

The PSORI-CM01 decoction reportedly inhibited mitosis of mouse vaginal epithelium significantly and promoted the formation of granular layers in mouse tail-scale epidermis compared with a saline group. It inhibited HaCaT cell growth after treatment for 24 h. The mechanism of PSORI-CM01 in treating psoriasis may be related to the promotion of granular cell growth and inhibition of epidermal cell proliferation [18]. A metabolomics approach to explore the in vivo mechanism of PSORI-CM01 used fasting urine samples from patients with consecutive intake mono-therapy after 0, 4, 8, and 12 weeks and was analyzed by Orthogonal Projection on Latent Structures Discriminant Analysis (OPLS-DA). Treatment with the PSORI-CM01 decoction for 12 weeks decreased the PASI scores. The positive effect of Optimized Yinxieling (PSORI-CM01) may be explained in terms of altered metabolism after intervention. Research revealed increased 12a-Hydroxy-3-oxocholadienic acid levels significantly after treatment [19].

Our study had a number of limitations. For the primary and secondary outcomes, we had a relatively small sample size that limited our ability to show statistical significance. Seasons have an important effect on the severity of psoriasis. Most psoriatic patients experienced remission in hot summer and aggravation in cold winter. Patients even recover to normal spontaneously in warm climate. This pilot study was carried out when the cold winter turned into hot summer. Therefore, the results might show a sequence bias. Two patients lost to follow-up because of unsatisfied efficacy were randomized to the CHM and placebo groups equally. Our intention-to-treat analysis with a last observation carried forward approach, may be associated with a withdrawal bias and bias associated with missed clinical data. Outcome measures such as the PASI and BSA scores were assessed by two investigators. Before commencing the study, the two investigators were trained in the evaluation of PASI together to develop similar levels of expertise in assessing the severity of erythema, desquamation, infiltration, and area of involvement. The same patient was assessed by the same investigators during the whole study. However, differences between the two investigators persisted due to the disadvantage of PASI scores, which were occasionally subjective. Therefore, in a full RCT, we should streamline the PASI training of the investigators responsible for outcome assessment, for consistency and quality of the primary outcome measure derived from PASI.

In this paper, we describe a pilot randomized controlled trial of oral Chinese medicine (PSORI-CM01) combined with topical sequential treatment for moderate-to-severe PV. The trial is outlined according to the CONSORT 2010 Checklist [20], and more details are shown in Additional file 1. The results show that it is feasible to develop the pilot trial into a full RCT. In this pilot study, the relapse rate of the placebo group was 67 %, different from the study by White et al. [9], which showed a relapse rate of 37.3 % with the same topical sequential therapy for 12 weeks. For the full RCT protocol [10], we have calculated the sample size based on the White et al. study. Supposing the relapse rate of PSORI-CM01 combined with topical sequential therapy for PV in the 12^th^ week was 20 %, the topical sequential therapy alone resulted in 37 %. We use the primary outcome measure to calculate sample size in the protocol by supposing that the addition of PSORI-CM01 reduced the relapse rate by 17 % more than the placebo. The pilot study showed that the relapse rate of the CHM group was 17 %, close to our hypothesis. The relapse rate decreased to 50 % in the CHM group compared with that in the placebo group, which exceeded our hypothesis and suggested that the sample size in the protocol was adequate.

Results from our placebo-controlled exploratory study added to the limited data on the combination of therapy with CHM and topical therapy in psoriasis patients, indicating that the addition of the PSORI-CM01 reduced the relapse rate compared with topical sequential therapy alone. PSORI-CM01 was safe and effective. Further study involving a larger patient population is warranted to evaluate the efficacy and safety of combining PSORI-CM01 and topical sequential therapy in patients with moderate-to-severe psoriasis, especially to confirm the decreased recurrence rate.

## Conclusion

The recurrence of PV is a therapeutic challenge for clinicians. Our research showed that PSORI-CM01, a Chinese herbal formula, combined with topical sequential therapy (calcipotriol betamethasone ointment daily for 4 weeks, followed by calcipotriol ointment daily for 8 weeks) resulted in a smaller recurrence rate than placebo combined with the same topical therapy. Our result indicates that the addition of PSORI-CM01 reduced the recurrence rate in moderate-to-severe psoriasis. Additional studies are needed using large sample sizes in a multicenter randomized control trial.

## Additional file

Additional file 1: CONSORT 2010 checklist for the manuscript. Page numbers of the manuscript were reported in the additional table to show the information according to the CONSORT 2010 checklist. (DOCX 18 kb)

## Abbreviations

BMI: body mass index
BSA: body surface area for PV
CHM: Chinese herbal medicine
CM: Chinese medicine
DLQI: Dermatology Life Quality Index
GPHCM: Guangdong Provincial Hospital of Chinese Medicine
HRQOL: health-related quality of life
KUMCR: Key Unit of Methodology in Clinical Research
PASI: Psoriasis Area Severity Index
PASI-50: PASI score decreases more than 50 % from baseline
PUVA: Psoralen combined with ultraviolet A
PV: psoriasis vulgaris
QOL: quality of life
SAEs: serious adverse events
SAS: self-rating anxiety scale
SDS: self-rating depression scale
SF-36: The MOS: Medical Outcomes Study item short form health survey
VAS: visual analog scale
