# Supplementary material for: Oral PSORI-CM01, a Chinese herbal formula, plus topical sequential therapy for moderate-to-severe psoriasis vulgaris: pilot study for a double-blind, randomized, placebo-controlled trial
Source: Trials. 2016 Mar 16;17:140. doi: 10.1186/s13063-016-1272-x (PMC4793560; doi:10.1186/s13063-016-1272-x)
Supplement: Additional file 1: — CONSORT 2010 checklist for the manuscript. Page numbers of the manuscript were reported in the additional table to show the information according to the CONSORT 2010 checklist. (DOCX 18 kb) [file 13063_2016_1272_MOESM1_ESM.docx]

**CONSORT 2010 checklist for the manuscript**

| \| Section/Topic \| **Item No** \| **Checklist item** \| \| --- \| --- \| --- \| | | \| Item No \| \| --- \| | | Checklist | \| **Reported on page No.** \| \| --- \| | | |
| --- | --- | --- | --- | --- | --- | --- | --- | --- | --- | --- | --- | --- |
| Title and abstract | | | | | | | |
|  | | | 1a | Identification as a randomised trial in the title | | Title Page | |
|  |  |  | 1b | Structured summary of trial design, methods, results, and conclusions (for specific guidance see CONSORT for abstracts | | Page 3 | |
| Introduction | | | | | | | |
|  | | | 2a | Scientific background and explanation of rationale | | Page 5-6 | |
|  |  |  | 2b | Specific objectives or hypotheses | | Page5;Page6, Paragraph 3 | |
| \| Methods \| \| --- \| | | | | | | | |
| Trial design | | | 3a | Description of trial design (such as parallel, factorial) including allocation ratio | | Page 6, section of Method,design and eligibility | |
|  |  |  | 3b | Important changes to methods after trial commencement (such as eligibility criteria), with reasons | | Page 7, section of Method, setting | |
| Participants | | | 4a | Eligibility criteria for participants | | Page 7, section of Method, patients,form Line 3 | |
|  |  |  | 4b | Settings and locations where the data were collected | | Page 7, Paragraph 3, Line 1 | |
| Interventions | | | 5 | The interventions for each group with sufficient details to allow replication, including how and when they were actually administered | | Page 8-9, section of Intervention | |
| Outcomes | | | 6a | Completely defined pre-specified primary and secondary outcome measures, including how and when they were assessed | | Page 9, section of Outcome measures | |
|  |  |  | 6b | Any changes to trial outcomes after the trial commenced, with reasons | | Not applicable | |
| Sample size | | | 7a | How sample size was determined | | Page 10, section of Sample size | |
|  |  |  | 7b | When applicable, explanation of any interim analyses and stopping guidelines | | Not applicable | |
| Randomisation: | | |  |  | |  | |
| Sequence  generation | | | 8a | Method used to generate the random allocation sequence | | Page 6, section of Method, design and eligibility | |
|  |  |  | 8b | Type of randomisation; details of any restriction (such as blocking and block size) | | Page 6, Paragraph 4, Line 4 | |
| Allocation concealment mechanism | | | 9 | Mechanism used to implement the random allocation sequence (such as sequentially numbered containers), describing any steps taken to conceal the sequence until interventions were assigned | | Page 6, the last line; Page 7, Paragraph 1, Line 1-3. | |
| Implementation | | | 10 | Who generated the random allocation sequence, who enrolled participants, and who assigned participants to interventions | | Page 6, section of Method,design and eligibility;Page 7 Paragraph 3, Line 1 | |
| Blinding | | | 11a | If done, who was blinded after assignment to interventions (for example, participants, care providers, those assessing outcomes) and how | | Page 7, Paragraph 1, Line 2-3. | |
|  |  |  | 11b | If relevant, description of the similarity of interventions | | Page 8, Paragraph 2,  Line 9-12 | |
| Statistical methods | | | 12a | Statistical methods used to compare groups for primary and secondary outcomes | | Page 9-10, section of Statistical analysis | |
|  |  |  | 12b | Methods for additional analyses, such as subgroup analyses and adjusted analyses | | Not appilicable | |
| Results | | | | | | | |
| Participant flow (a diagram is strongly recommended) | 13a | | | For each group, the numbers of participants who were randomly assigned, received intended treatment, and were analysed for the primary outcome | | | Figure 1; Page 10, the last paragraph |
|  | 13b | | | For each group, losses and exclusions after randomisation, together with reasons | | | Figure 1; Page 10, the last paragraph |
| Recruitment | 14a | | | Dates defining the periods of recruitment and follow-up | | | Page 10, the last paragraph, Line 1 |
|  | 14b | | | Why the trial ended or was stopped | | | Not applicable |
| Baseline data | 15 | | | A table showing baseline demographic and clinical characteristics for each group | | | Table 1 |
| Numbers analysed | 16 | | | For each group, number of participants (denominator) included in each analysis and whether the analysis was by original assigned groups | | | Figure 1. Table 2 and Table 3 |
| Outcomes and estimation | 17a | | | For each primary and secondary outcome, results for each group, and the estimated effect size and its precision (such as 95% confidence interval) | | | Table 2 and Table 3.  Figure 2 and Figure 3 |
|  | 17b | | | For binary outcomes, presentation of both absolute and relative effect sizes is recommended. | | | Table 2 |
| Ancillary analyses | 18 | | | Results of any other analyses performed, including subgroup analyses and adjusted analyses, distinguishing pre-specified from exploratory | | | Not applicable |
| Harms | 19 | | | All important harms or unintended effects in each group (for specific guidance see CONSORT for harms) | | | Page 12-13, section of Safety |
| Discussion | | | | | | | |
| Limitations | 20 | | | Trial limitations, addressing sources of potential bias, imprecision, and, if relevant, multiplicity of analyses | | | Page 15, Paragraph 2 |
| Generalisability | 21 | | | Generalisability (external validity, applicability) of the trial findings | | | Page 13, Paragraph 3 |
| Interpretation | 22 | | | Interpretation consistent with results, balancing benefits and harms, and considering other relevant evidence | | | Page 14, Paragraph 2-4; Page 15, Paragraph 1 |
| Other information | | | | | | |  |
| Registration | 23 | | | Registration number and name of trial registry | | | Page 3-4 |
| Protocol | 24 | | | Where the full trial protocol can be accessed, if available | | | Page 21, Citation 10. |
| Funding | 25 | | | Sources of funding and other support (such as supply of drugs), role of funders | | | Page 19 |
